# CD147 regulates cancer migration *via* direct interaction with Annexin A2 and DOCK3-β-catenin-WAVE2 signaling

**DOI:** 10.18632/oncotarget.6723

**Published:** 2015-12-22

**Authors:** Hong-Yong Cui, Shi-Jie Wang, Ji-Yu Miao, Zhi-Guang Fu, Fei Feng, Jiao Wu, Xiang-Min Yang, Zhi-Nan Chen, Jian-Li Jiang

**Affiliations:** ^1^ Cell Engineering Research Center and Department of Cell Biology, State Key Laboratory of Cancer Biology, National Key Discipline of Cell Biology, Fourth Military Medical University, Xi'an, P.R. China

**Keywords:** CD147, Annexin A2, DOCK3, WAVE2, cell movement

## Abstract

The acquisition of inappropriate migratory feature is crucial for tumor metastasis. It has been suggested that CD147 and Annexin A2 are involved in regulating tumor cell movement, while the regulatory mechanisms are far from clear. In this study, we demonstrated that CD147 physically interacted with the N-terminal domain of Annexin A2 and decreased Annexin A2 phosphorylation on tyrosine 23. *In vitro* kinase assay showed that the I domain of CD147 was indispensable for CD147-mediated downregulation of Annexin A2 phosphorylation by Src. Furthermore, we determined that p-Annexin A2 promoted the expression of dedicator of cytokinesis 3 (DOCK3) and DOCK3 blocked β-catenin nuclear translocation, resulting in inhibition of β-catenin signaling. In addition, DOCK3 inhibited lamellipodium dynamics and tumor cell movement. Also, we found that β-catenin signaling increased WAVE2 expression. Therefore, DOCK3 was characterized as a negative regulator of WAVE2 expression via inhibiting β-catenin signaling. Our study provides the first evidence that CD147 promotes tumor cell movement and metastasis via direct interaction with Annexin A2 and DOCK3-β-catenin-WAVE2 signaling axis.

## INTRODUCTION

The majority of deaths associated with cancer are due to the metastasis of the original tumor cells [1]. Metastasis is an exceedingly complex and multi-step process. The acquisition of inappropriate migratory and invasive characteristics is a common feature of metastatic cancer cells. Rho family GTPases are intracellular signaling molecules that play critical roles in regulating cytoskeleton reorganization and cell movement [2]. The activities of most Rho family members depend on a delicate balance between the GTP-bound, active state and the GDP-bound, inactive state. The cycling between these two states is positively controlled by guanine nucleotide exchange factors (GEFs).

CD147 is a type I transmembrane glycoprotein. The extracellular portion of CD147 consists of an N-terminal IgC2 domain and a C-terminal IgI domain [3]. Previously, we reported that CD147 coprecipitates and colocalizes with Annexin A2 in hepatocellular carcinoma (HCC) cells and they may form a functional complex [4]. We also found that depletion of CD147 resulted in a rounded morphology, whereas the depletion of Annexin A2 produced an elongated morphology. CD147 may inhibit RhoA/ROCK signaling pathway by inhibiting Annexin A2 phosphorylation [5]. However, the evidence to support the direct interaction between CD147 and Annexin A2 is still absent and the mechanisms by which CD147 inhibits Annexin A2 phosphorylation and regulates RhoA activity are far from clear.

Annexin A2 was first identified as a major substrate of v-Src [6]. The N-terminus possesses a phosphorylation sites on tyrosine 23 which is a substrate for phosphorylation by Src [7], serine 25 which has been reported to be phosphorylated by protein kinase C [8] and serine 11 which can be phosphorylated by protein kinase A [9]. Annexin A2 phosphorylation on tyrosine 23 plays an essential role in regulating cofilin-dependent actin cytoskeleton dynamics [10]. Recently, it is reported that cyclic adenosine monophosphate-induced von Willebrand factor secretion is promoted by serine 11 phosphorylation of Annexin A2, which is dephosphorylated *via* calcineurin-like phosphatase [9]. However, the negative regulation of Annexin A2 tyrosine phosphorylation is not well known.

DOCK3, also known as modifier of cell adhesion (MOCA), is one member of the DOCK GEF family. DOCK3 was originally identified as being one of the presenilin-binding proteins [11], is expressed in neuronal tissues, and is involved in cell adhesion and axonal degeneration [12, 13]. DOCK3 is also described as critical for axonal outgrowth stimulating both actin rearrangement and microtubule assembly pathways [14, 15]. In parallel of its neuronal functions, DOCK3 is also expressed in non-neuronal cells. Sanz-Moreno et al showed that suppression of DOCK3 blocks the transition between amoeboid and mesenchymal movement [16]. Ladhani et al identified DOCK3 as a critical regulator of Rac1 activity [17]. All these results indicate that DOCK3 regulates cytoskeleton rearrangement mainly *via* its GEF activity, however, whether DOCK3 could regulate cell movement independent of its GEF activity and its regulation in cancer cells are mainly unknown.

Here, we demonstrated that the I domain of CD147 interacts directly with the N-terminal domain of Annexin A2, and this interaction inhibits c-Src phosphorylation of Annexin A2 on tyrosine 23. Furthermore, DOCK3 is regulated by Annexin A2 phosphorylation and negatively regulates WAVE2 expression as an inhibitor of β-catenin signaling.

## RESULTS

### The Ig-like domains of CD147 interact directly with Annexin A2

**Figure 1:**
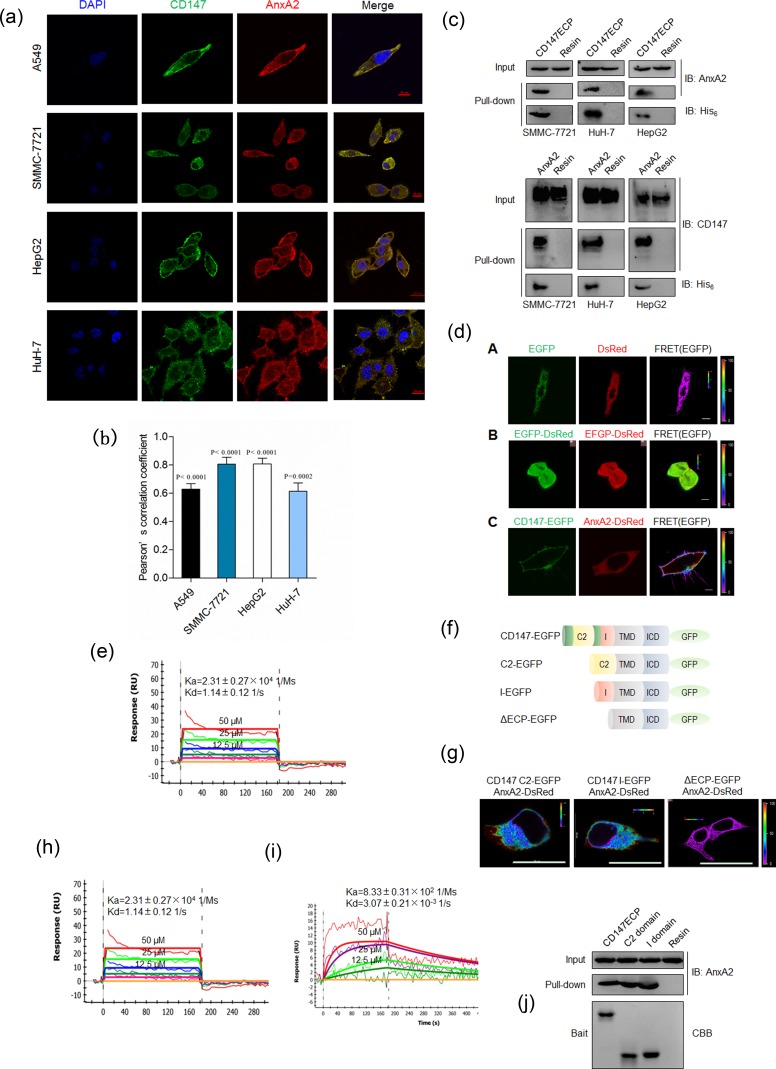
CD147 physically interacts with Annexin A2 **a.** Colocalization of CD147 and Annexin A2 in indicated cells. Scale bar, 10μm (A549 cells) or 20μm (other cells). **b.** Quantitative colocalization analysis of localization of CD147 and Annexin A2. The PCC values were calculated with NIS-Elements software (Nikon, Japan). The student's *t*-test was used to test the significance of PCC measurements of colocalization. **c.** Pull-down assay for CD147ECP-Annexin A2 interaction. **d.** The interaction between CD147 and Annexin A2 was analyzed with FRET. The color bar represents FRET ratio. Scale bar, 10μm. **e.** Biophysical analysis of the CD147ECP-Annexin A2 interaction using SPR. A concentration series of purified CD147ECP were injected over immobilized Annexin A2, and biophysical parameters derived from a 1:1 binding model. RU, response units. **f.** A schematic illustration of the structures used in FRET imaging. C2, C2 domain; I, I domain; TMD, transmembrane domain; ICD, intracellular domain. **g.** Interaction between the indicated constructs was analyzed with FRET. The color bar represents FRET ratio. Scale bar, 20μm. **h.**-**i.** Biophysical analysis of CD147 C2 domain-Annexin A2 interaction (h) and CD147 I domain-Annexin A2 interaction (i) using SPR. RU, response units. j Pull-down of endogenous Annexin A2 with purified constructs of human CD147. CBB, coomassie brilliant blue.

We first determined the cellular localization of CD147 and Annexin A2. As shown in Figure 1a, CD147 and Annexin A2 were distributed in membrane and cytoplasm in three HCC cell lines and lung cancer A549 cells. Colocalization analysis based on Pearson's correlation coefficient (PCC) showed that there was a high degree of colocalization between CD147 and Annexin A2 (Figure 1b). We previously also performed co-localization assay using ER-Tracker to indicate ER location and we found that Annexin A2 Y23F co-localized with CD147 in cytoplasm closed to ER [5]. Also, we performed a His-tag pull-down assay to confirm the interaction between CD147 and Annexin A2. As shown in Figure 1c, purified His-tagged Ig-like domains of CD147 (CD147ECP-his) could capture Annexin A2 expressed in HCC cells, and vice versa. These data indicated that CD147 may interact with Annexin A2.

To test whether CD147 interacts directly with Annexin A2 in living cells, fluorescence resonance energy transfer (FRET) assay was chosen to analyze the interaction. EGFP and DsRed were tagged to CD147 and Annexin A2, respectively. The ability and efficiency of CD147 and Annexin A2 to interact with each other were evaluated by transfecting cells with the FRET pair CD147-EGFP/Annexin A2-DsRed. As shown in Figure 1d, use of the CD147-EGFP/Annexin A2-DsRed FRET pair yielded FRET efficiency significantly higher than that obtained with the negative control, where the tandem expression of EGFP and DsRed was used as positive control, which demonstrated that there is a direct interaction between CD147 and Annexin A2. We then characterized the kinetic binding parameters using purified proteins and SPR and showed that the Ig-like domains of CD147 interacted directly with Annexin A2 (Figure 1e). These parameters are typical of extracellular protein interactions measured using this technique [18].

As CD147 is known to contain two Ig-like domains [19], we then truncated CD147 (Figure 1f) and examined which Ig-like domain of CD147 was responsible for the interaction with Annexin A2. As shown in Figure 1g, both Ig-like domains of CD147 interacted with Annexin A2 as efficiently as CD147ECP. The binding parameters were also determined (Figure 1h-1i). Consistent with this, purified C2 domain and I domain could capture Annexin A2 expressed in HCC cells, respectively (Figure 1j).

### CD147 down-regulates Annexin A2 phosphorylation

**Figure 2:**
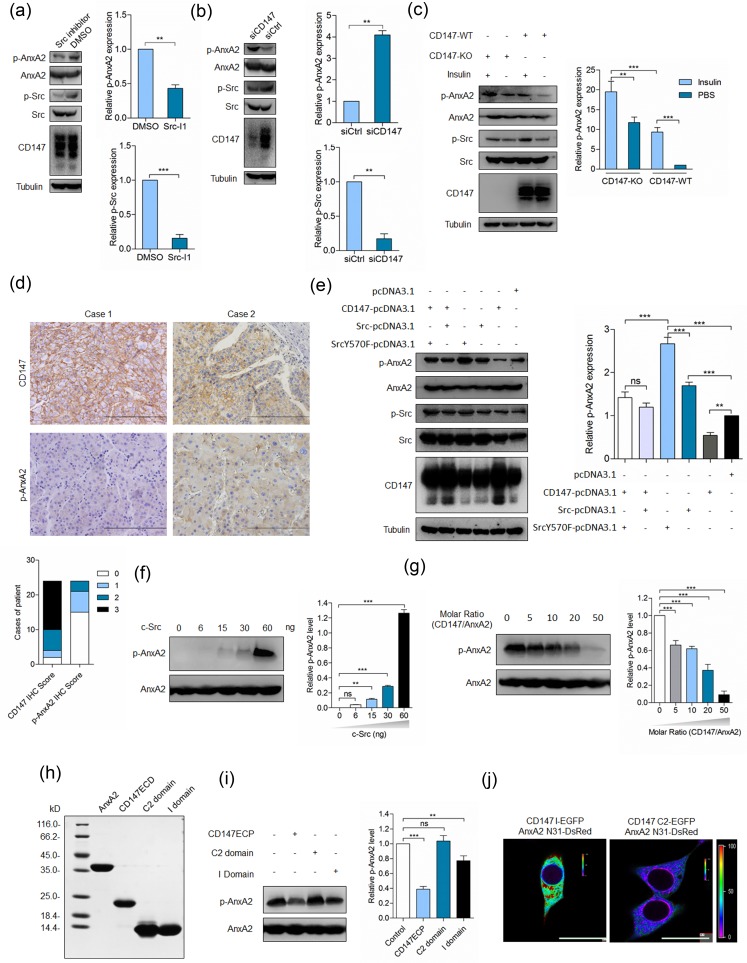
CD147 inhibits Annexin A2 phosphorylation by Src **a.** SMMC-7721 cells were treated with Src inhibitor (Src I-1) for 24 h and analyzed for p-Src and p-Annexin A2. ***p < 0.001, **p < 0.01 by student's *t*-test. **b.** SMMC-7721 cells were transfected with a pool of siRNAs targeting CD147 (siCD147) or control siRNA (siCtrl) and analyzed for p-Src and p-Annexin A2 at 48 h after transfection. **p < 0.01 by student's *t*-test. **c.** Total cell lysates of cells in the indicated conditions were analyzed by Western blot. ***p < 0.001, **p < 0.01 by ANOVA. **d.** Representative images of the IHC staining. Scale bars, 200 μm. **e.** Total cell lysates of cells transfected with indicated constructs were analyzed by Western blot at 48 h after transfection. ***p < 0.001, **p < 0.01, ns *p* > 0.05 by ANOVA. **g.** Src phosphorylates Annexin A2 *in vitro*. Indicated amounts of purified Src proteins were added to the reactions. The phosphorylation level of Annexin A2 was determined by Western blot. ***p < 0.001, **p < 0.01, ns *p* > 0.05 by ANOVA. **g.** A concentration series of purified CD147ECP was added to the reactions where Src was 60ng. The phosphorylation level of Annexin A2 was determined by Western blot. ***p < 0.001 by ANOVA. **h.** Coomassie blue staining of purified proteins used in the *in vitro* kinase assay. **i.** Indicated proteins were added to the reactions and probed with phospho-specific antibody. ***p < 0.001, **p < 0.01, ns *p* > 0.05 by ANOVA. **j.** Interaction between the indicated constructs was analyzed with FRET. The color bar represents FRET ratio. Scale bar, 20 μm. Error bars indicate the standard deviations (SD) from at least triplicate determinations (*n* > 3)

Annexin A2 is a substrate of Src and its phosphorylation by Src would induce multiple effects [10, 20, 21]. We found that Src inhibitor could downregulate Annexin A2 phosphorylation (Figure 2a), indicating that Src could phosphorylate Annexin A2 in cancer cells. Previously, we have revealed that CD147 upregulates Src activity *via* FAK [22], however, Tyr23 phosphorylation of Annexin A2 was negatively related to the expression of CD147 [5], which was confirmed as in Figure 2b. We found that insulin treatment could upregulate Src activity and Annexin A2 phosphorylation on Tyr23, however, elevated Annexin A2 phosphorylation induced by insulin treatment in K-7721 cells was attenuated in SMMC-7721 cells (Figure 2c). We also evaluated CD147 expression and Annexin A2 phosphorylation in HCC tissues. High CD147 expression was observed in 83.3% (20/24) of HCC tissues, while Annexin A2 phosphorylation on Tyr23 was below detectable level in 62.5% (15/24) of HCC tissues (Figure 2d). Pearson's correlation coefficient calculated between staining intensity for p-AnxA2 and CD147 was −0.437 (*p* = 0.033). To better understand this regulation, SMMC-7721 cells were transfected with plasmids to express CD147 alone or in the presence of ectopically-expressed wild-type (WT) Src or dominant positive mutant (SrcY570F). As shown in Figure 2e and ESM_1, Annexin A2 phosphorylation was increased in cells expressing WT Src or SrcY570F, which was attenuated in cells overexpressing CD147 together with WT Src or SrcY570F, indicating that Annexin A2 can be phosphorylated by Src and this phosphorylation can be attenuated, at least partially, by CD147.

### Interaction with CD147 inhibits Annexin A2 phosphorylation by Src

To address whether CD147 interacting with Annexin A2 inhibited Annexin A2 phosphorylation by Src, an *in vitro* kinase assay was performed. Purified Annexin A2 was resuspended in kinase buffer, and incubated in the presence of ATP and Src. As expected, the phosphorylation level of Annexin A2 was increased with the increase of Src in the reaction system when the reaction was blotted with anti-p-Annexin A2 antibody (Figure 2f). However, when purified CD147ECP was added to the reaction system, we found that the phosphorylation level of Annexin A2 was dramatically decreased (Figure 2g), indicating that CD147 could inhibit Annexin A2 phosphorylation by Src *in vitro*.

As we have revealed that both Ig-like domains of CD147 could interact with Annexin A2, we supposed that both domains also could inhibit Annexin A2 phosphorylation when added into the reaction system. We purified I domain and C2 domain and the purity was determined by coomassie blue staining (Figure 2h). Strikingly, we found that I domain, but not C2 domain, was able to at least partially inhibit Annexin A2 phosphorylation (Figure 2i). Given that the tyrosine phosphorylation was occurred at the N-terminal domain of Annexin A2, we determined the interaction between I domain of CD147 and the N-terminal domain of Annexin A2. We found that I domain but not C2 domain could interact directly with the N-terminal domain of Annexin A2 in living cells (Figure 2j). Considering that I domain of CD147 interacts with Annexin A2 and exerts inhibitory effects on Annexin A2 phosphorylation and CD147 interacts with phosphorylation-inactive mutant of Annexin A2 but not with phospho-mimicking mutant of Annexin A2 [5], it may be a plausible explanation that this direct interaction inhibits Annexin A2 phosphorylation by Src. Taken together, these results demonstrate that I domain is necessary for CD147-mediated downregulation of Annexin A2 phosphorylation.

### Annexin A2 phosphorylation regulates DOCK3 expression

**Figure 3:**
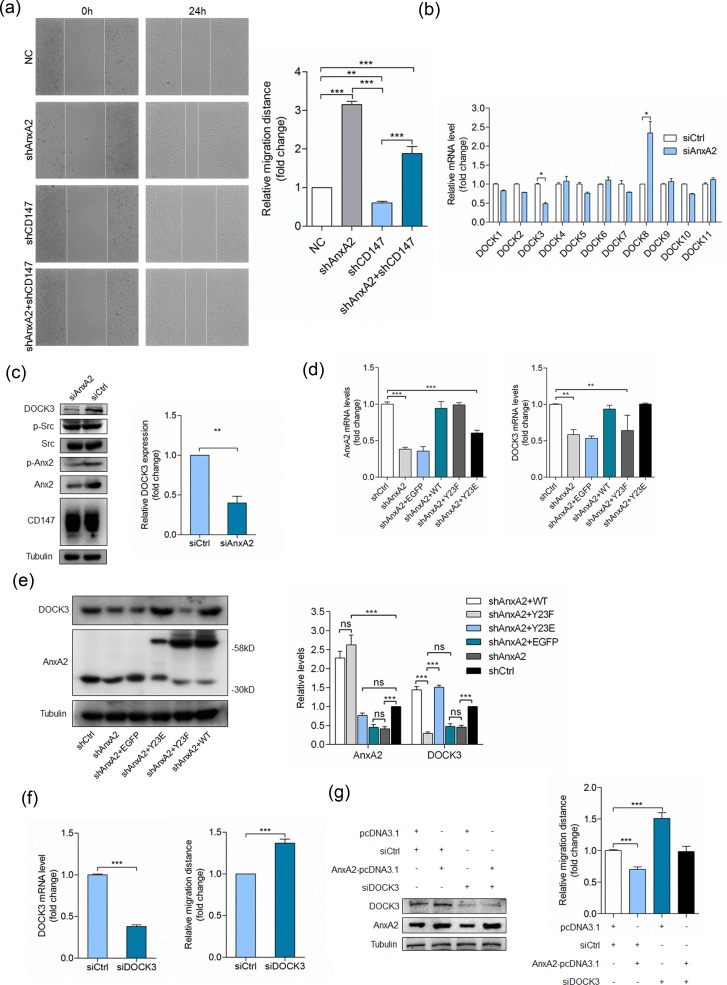
Annexin A2 phosphorylation regulates cell movement *via* DOCK3 **a.** Quantitative analysis of relative migration distance. ***p < 0.001, **p < 0.01 by ANOVA. **b.** Relative quantitative real-time RT-PCR analysis of DOCK family GEFs in HuH-7 cells transfected with a pool of siRNAs targeting Annexin A2 (siAnxA2) or control siRNA (siCtrl) at 24 h after transfection, after normalization to the expression level with siCtrl transfection. *p < 0.05 by student's *t*-test. **c.** HuH-7 cells were transfected with siAnxA2 or siCtrl and analyzed for DOCK3 by Western blot at 48 h after transfection. **p < 0.01 by student's *t*-test. **d.** Relative quantitative real-time RT-PCR analysis of Annexin A2 (left pannel) and DOCK3 (right panel) in HuH-7 cells transfected with indicated constructs at 24 h after transfection, after normalization to the expression level with scramble shRNA transfection. ***p < 0.001, **p < 0.01 by ANOVA. **e.** HuH-7 cells transfected with indicated constructs were analyzed for Annexin A2 and DOCK3 by Western blot at 48 h after transfection. ***p < 0.001, ns *p* > 0.05 by ANOVA. **f.** HuH-7 cells transfected with a pool of siRNAs targeting DOCK3 (siDOCK3) or siCtrl were analyzed for DOCK3 by relative quantitative real-time RT-PCR (left panel) and relative migration distance by wound-healing assay (right panel) at 24 h after transfection. ***p < 0.001 by student's *t*-test. **g.** HuH-7 cells transfected with indicated constructs were analyzed for DOCK3 and Annexin A2 by Western blot (left panel) at 48 h after transfection and relative migration distance by wound-healing assay (right panel) at 24 h after transfection. ***p < 0.001 by ANOVA. Error bars indicate the standard deviations (SD) from at least triplicate determinations (*n* > 3)

Annexin A2 phosphorylation could regulate Rho-mediated actin rearrangement and cell adhesion [23-25]. Consistent with previous studies [26-28], we found that silencing Annexin A2 significantly enhanced cell movement (Figure 3a). Silencing CD147 inhibited cell movement, while silencing Annexin A2 simultaneously could reverse the inhibitory effect (Figure 3a), indicating that CD147 may regulate cell movement through Annexin A2. To determine the molecular mechanisms underlying cell movement regulation by Annexin A2, we transfected SMMC-7721 cells with a pool of siRNAs targeting Annexin A2, and detected the mRNA level of DOCK family GEFs. Consistent with our previous study [29], we found that silencing Annexin A2 led to DOCK3 downregulation and DOCK8 upregulation (Figure 3b). The protein expression was also verified by Western blot (Figure 3c). These results indicated that DOCK3 as a GEF may be involved in cell movement regulation by Annexin A2. Next we established an Annexin A2 stable knockdown cell line HuH-7 shA2 using lentivirus. HuH-7 shA2 cells were transfected with RNA interference-resistant plasmids to express wide-type Annexin A2 (WT), phosphorylation inactive mutant (Y23F), or phosphorylation mimic mutant (Y23E). As shown in Figure 3d, the expression of Annexin A2 in WT and Y23F cells was restored to that of the parent cells and the expression of Annexin A2 in Y23E cells was partially restored. However, the expression of DOCK3 in Y23E cells was fully restored and the phosphorylation inactive mutant Y23F failed to restore DOCK3 expression (Figure 3d). The protein expression level was also verified by Western blot (Figure 3e). These results demonstrated that Annexin A2 promoting DOCK3 expression is tyrosine phosphorylation dependent.

### DOCK3 is required for Annexin A2 phosphorylation to regulate cell movement

To investigate whether Annexin A2 phosphorylation regulates cell movement *via* DOCK3, we first evaluated the effects of DOCK3 knockdown on cell motility. Figure 3f showed that silencing DOCK3 led to increased cell movement, similar to that of silencing Annexin A2. We then examined whether the increased DOCK3 contributed to the reduced cell motility in Annexin A2 overexpressing cells. This hypothesis predicted that reducing DOCK3 should decrease the impact of Annexin A2 overexpression on cell motility. Accordingly, we transfected HuH-7 cells with Annexin A2-pcDNA3.1, and then selectively depleted DOCK3 by a pool of three siRNAs (Figure 3g). Cell motility was reduced in cells transfected with Annexin A2-pcDNA3.1 together with control siRNAs. Strikingly, however, Annexin A2 overexpression did not reduce cell motility to the same level in DOCK3 knockdown cells as it did in control cells. This implies that DOCK3 contributes to the regulation of cell movement by Annexin A2 phosphorylation.

### Silencing of DOCK3 increases cell motility by promoting WAVE2 expression

**Figure 4:**
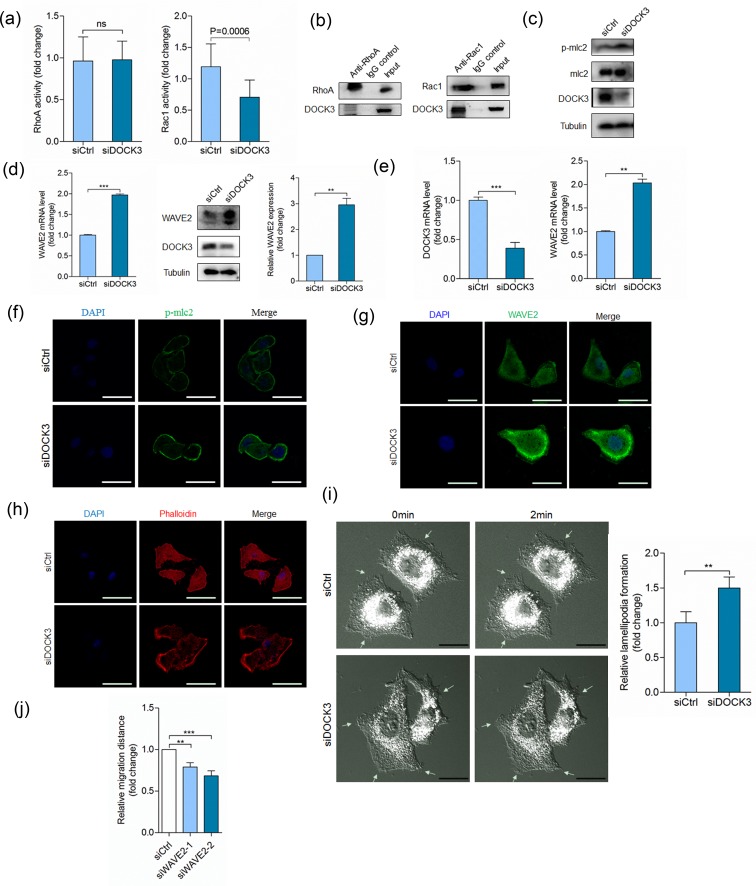
DOCK3 inhibits WAVE2 expression **a.** Quantification of RhoA (left panel) and Rac1 (right panel) activation in HuH-7 cells transfected with siDOCK3 or siCtrl at 48 h after transfection. ns *p* > 0.05 by student's *t*-test. **b.** Co-immunoprecipitation of endogenous RhoA (left panel) or Rac1 (right panel) with DOCK3. **c.** HuH-7 cells transfected with siDOCK3 or siCtrl were analyzed for p-mlc2 and DOCK3 by Western blot at 48 h after transfection. **d.** HuH-7 cells transfected with siDOCK3 or siCtrl were analyzed for WAVE2 by relative quantitative real-time RT-PCR (left panel) and Western blot (right panel) at 48 h after transfection. ***p < 0.001 by student's *t*-test. **e.** Relative quantitative real-time RT-PCR analysis of DOCK3 (left panel) and WAVE2 (right panel) in A549 cells transfected with siDOCK3 or siCtrl at 48 h after transfection. ***p < 0.001, **p < 0.01 by student's *t*-test. **f.**-**h.** Confocal microscopy images of p-mlc2 expression (f), WAVE2 expression (g) and actin cytoskeleton rearrangement (h) in A549 cells transfected with siDOCK3 or siCtrl at 48 h after transfection. **i.** DIC microscopy images of A549 cells transfected with siDOCK3 or siCtrl at 48 h after transfection. Arrows indicate lamellipodium dynamics. **p < 0.01 by student's *t*-test. **j.** Quantitative analysis of relative migration distance. ***p < 0.001, **p < 0.01 by ANOVA. Error bars indicate the standard deviations (SD) from at least triplicate determinations (*n* > 3)

DOCK3 has been previously characterized as a GEF for Rac1 [30], therefore, we investigated whether DOCK3 regulates cell movement by influencing Rac1 activity. Silencing DOCK3 reduced Rac1 activity, which had no effect on RhoA activity (Figure 4a). Also DOCK3 could immunoprecipitate with Rac1 and negligible amount of RhoA (Figure 4b). Also, we detected the effector molecules downstream of Rac1 and RhoA signaling pathways. DOCK3 knockdown increased phosphorylation level of mlc2 (Figure 4c), however, DOCK3 knockdown strikingly increased WAVE2 expression at the mRNA and protein levels (Figure 4d). We recapitulated this effect in A549 cells (Figure 4e). Immunofluorescence staining showed that silencing DOCK3 led to increased mlc2 phosphorylation and WAVE2 expression (Figure 4f-4g). These results indicated that DOCK3-mediated cell movement regulation is not mainly mediated by Rac1 signaling, thus independent of its guanine nucleotide exchange activity. Furthermore, we evaluated the effects of DOCK3 knockdown on cytoskeleton rearrangement. As shown in Figure 4h, silencing of DOCK3 increased lamellipodium formation. Live-cell imaging also showed that silencing of DOCK3 increased lamellipodium formation (ESM_2-3 and Figure 4i). We also found that silencing WAVE2 led to decreased cell motility (Figure 4j). Thus, increased cell movement induced by depletion of DOCK3 may due to increased WAVE2 expression and lamellipodium formation.

### DOCK3 regulates WAVE2 expression as a negative regulator of β-catenin signaling

**Figure 5:**
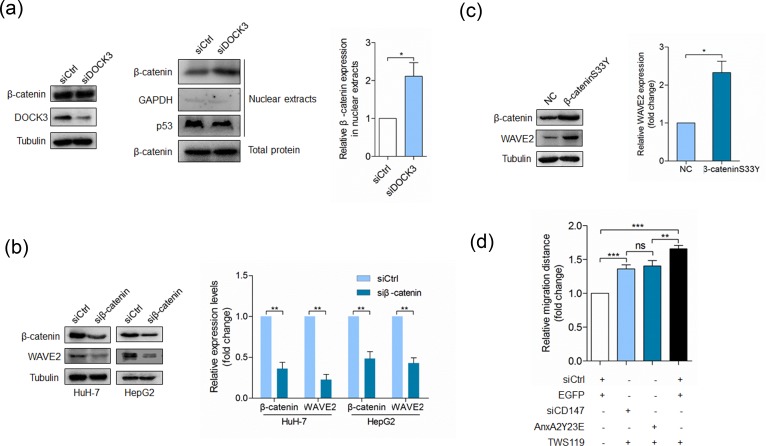
DOCK3 inhibits WAVE2 expression as a negative regulator of β-catenin signaling **a.** Western blot analysis for β-catenin and DOCK3 at 48 h after transfection with siDOCK3 or siCtrl (left panel). Western blot analysis for β-catenin, p53 and GAPDH in nuclear extracts at 48 h after transfection with siDOCK3 or siCtrl (middle panel). Quantitative analysis of nuclear-total ratio of β-catenin (right panel) *p* < 0.05 by student's *t*-test. **b.** Western blot analysis for β-catenin and WAVE2 in cells transfected with a pool of siRNAs targeting β-catenin (siβ-catenin) or siCtrl at 48 h after transfection. **p < 0.01 by student's *t*-test. **c.** Western blot analysis for WAVE2 in HuH-7 cells transfected with pcDNA3 or β-cateninS33Y-pcDNA3 at 48 h after transfection. *p < 0.05 by student's *t*-test. **d.** Quantitative analysis of relative migration distance. ***p < 0.001, **p < 0.01, ns *p* > 0.05 by ANOVA. Error bars indicate the standard deviations (SD) from at least triplicate determinations (*n* > 3)

We next determined the mechanism by which DOCK3 regulated WAVE2. As DOCK3 has been reported to form a complex with β-catenin and inhibit β-catenin activation [31], we wondered whether DOCK3 regulated WAVE2 expression *via* inhibiting β-catenin signaling. We examined the effect of DOCK3 knockdown on the localization of β-catenin. Consistent with the previous results, silencing of DOCK3 resulted in an increase in nuclear levels of β-catenin but did not affect the total amount of β-catenin (Figure 5a), indicating that DOCK3 affects the subcellular distribution of β-catenin. The effect of β-catenin activation on WAVE2 expression was evaluated in HuH-7 cells. Results shown in Figure 5b demonstrated that silencing of β-catenin led to reduced WAVE2 expression. Overexpression of a dominant active β-catenin mutant resulted in an increase of WAVE2 expression (Figure 5c). All these results argue that β-catenin activation promotes WAVE2 expression and this promotion can be repressed by DOCK3. We also evaluated the effects of GSK-3β inhibitor TWS119 on cell motility and found that TWS119 treatment could rescue the effects of Y23E overexpression or CD147 silencing on cell motility (Figure 5d).

### CD147 down-regulates DOCK3 expression *via* inhibiting Annexin A2 phosphorylation

**Figure 6:**
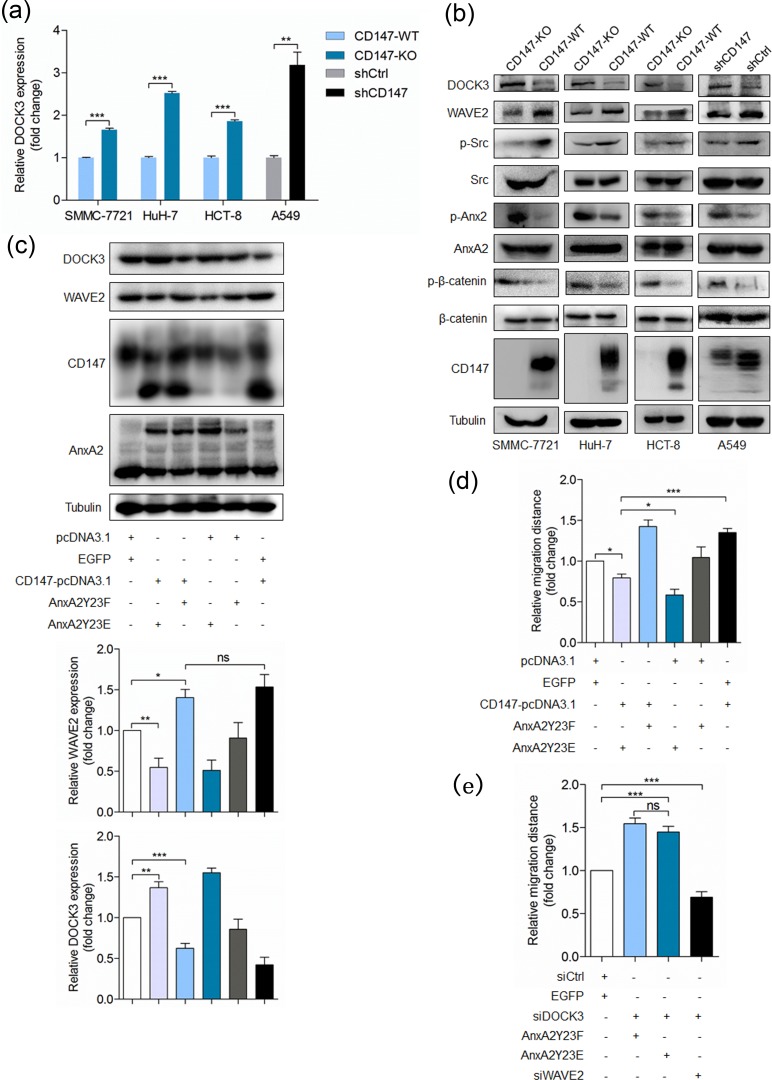
CD147 promotes cancer cell migration *via* inhibiting Annexin A2 phosphorylation and DOCK3 expression **a.** Relative quantitative real-time RT-PCR analysis of DOCK3 in indicated cell lines. ***p < 0.001, **p < 0.01 by student's *t*-test. **b.** Western blot analysis for DOCK3, WAVE2, p-Src, p-β-catenin and p-Annexin A2 in indicated cells. **c.** Western blot analysis for DOCK3 and WAVE2 in cells transfected with indicated constructs. ***p < 0.001, **p < 0.01, *p < 0.05, ns *p* > 0.05 by ANOVA. **d.**-**e.** Quantitative analysis of relative migration distance. ***p < 0.001, **p < 0.01, *p < 0.05, ns *p* > 0.05 by ANOVA. Error bars indicate the standard deviations (SD) from at least triplicate determinations (*n* > 3)

We have shown that CD147 inhibits Annexin A2 phosphorylation and Annexin A2 phosphorylation induces DOCK3 expression, thus we supposed that CD147 could inhibit DOCK3 expression and promote cell movement *via* interacting with Annexin A2. As expected, depletion of CD147 led to DOCK3 upregulation in indicated cell lines (Figure 6a-6b), indicating that CD147 can regulate DOCK3 expression. To further confirm the regulation mechanisms presented in this study, we also detected the other key molecules. We found that CD147 deletion resulted in increased Annexin A2 phosphorylation and decreased WAVE2 expression and Src activation (Figure 6b). Overexpression of phosphorylation mimic mutant Y23E dramatically attenuated the effects of CD147 overexpression on DOCK3 and WAVE2 expression (Figure 6c) and cell motility (Figure 6d). Furthermore, WAVE2 silencing rescued the effect of DOCK3 knockdown on cell motility and DOCK3 silencing rescued the effect of Y23E overexpression on cell motility (Figure 6e). Taken together, these results demonstrated that CD147 can promote cell movement *via* interacting with Annexin A2 and reducing DOCK3 expression, resulting in increased WAVE2 expression.

### CD147 promotes HCC metastasis in nude mice

Cytoskeletal rearrangement and cell motility achieve dire significance during tumor metastasis. We investigated whether CD147 influenced tumor metastasis *in vivo*. The incidence of metastasis after intra-splenic injection of SMMC-7721 or K-7721 cells was shown in Table 1. The metastasis was substantially suppressed in K-7721 group (*p* < 0.05) compared with that in SMMC-7721 group. As shown in Table 1 and Figure 7a, injection of SMMC-7721 cells into spleen resulted in 6 of 8 mice developing more than 50 visible liver metastases. However, only 1 of 8 mice in K-7721 group developed more than 50 visible liver metastases. IHC staining showed CD147 suppressed DOCK3 expression and enhanced WAVE2 expression (Figure 7a).

**Table 1:** Number of mice with metastasis in each group

No. Metastasis in Each Mouse	CD147-WT, *n* (%)	CD147-KO, *n* (%)^#^
<50	2 (25%)	7 (87.5%)
>50	6 (75%)	1 (12.5%)
Total	8	8

# CD147-KO versus CD147-WT: *p* < 0.05

**Figure 7:**
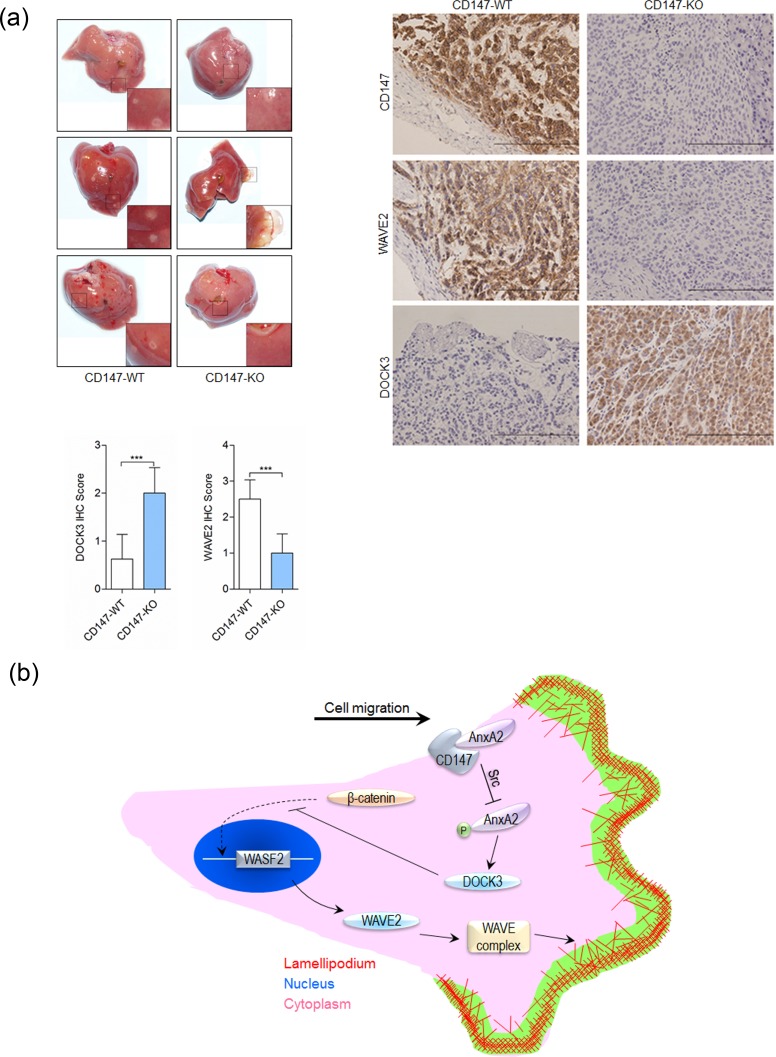
CD147 promotes cancer metastasis **a.** Metastasis assay by intra-splenic injection of SMMC-7721 or K-7721 cells in nude mice. Livers were excised for examination (left panel). Tumors were assessed by histology (right panel). Expression of CD147, WAVE2 and DOCK3 were examined by immunohistochemistry. Scale bar, 200μm. ***p < 0.001 by student's *t*-test. **b.** Schematic representation of the major mechanisms of CD147 in regulating cancer cell movement *via* interaction with Annexin A2. mlc2: myosin light chain 2; DOCK3: dedicator of cytokinesis 3; WAVE2: Wiskott-Aldrich syndrome family verprolin-homologous protein 2.

## DISCUSSION

In this study, we found that the I domain of CD147 could bind the N-terminal domain of Annexin A2 and inhibit Annexin A2 phosphorylation on tyrosine 23 by Src. Phosphorylation of Annexin A2 promoted the expression of DOCK3 and DOCK3 suppressed the expression of WAVE2 *via* inhibiting β-catenin signaling. Thus CD147 promoted cell movement *via* p-Annexin A2-DOCK3-β-catenin-WAVE2 signaling axis (Figure 7b).

One of the cellular functions of Annexin A2 is its involvement in cytoskeletal rearrangement, which is thought to be mediated by its tyrosine phosphorylation and its regulation of RhoA [5, 10, 23], indicating that Annexin A2 seems to regulate actin remodeling by acting upstream of RhoA. Whether Annexin A2 regulates Rac activity is not clear. Previous studies suggest that DOCK3 functions mainly dependent on its GEF catalytic activity in regulating cytoskeletal reorganization. We identified DOCK3 as a tyrosine phosphorylated Annexin A2-regulated RacGEF (Figure 4), we therefore hypothesized that p-Annexin A2 may regulate cytoskeleton rearrangement through DOCK3-mediated activation of Rac1 signaling. Consistent with previous studies, DOCK3 co-precipitates with Rac1 and silencing DOCK3 leads to reduced Rac1 activation. However, WAVE2, generally considered as a downstream effector of Rac1 signaling [32, 33], is dramatically increased both at mRNA and protein levels, indicating that DOCK3 may regulate WAVE2 independent of its guanine nucleotide exchange activity and Rac1 signaling. It may be plausible that the down-regulation of WAVE2 resulted from Rac1 inhibition can be greatly offset by the up-regulation of WAVE2 resulted from DOCK3 silence, thus leading to decreased Rac1 activity while increased WAVE2 expression. We also found that Annexin A2 suppressed DOCK8 expression, indicating that DOCK8 may be involved in Annexin A2-regulated cancer migration and progression.

CD147 has been reported to play important roles in cellular processes of HCC progression, including adhesion, migration and invasion [34-36]. Of note, CD147 interacts with integrin and increases α3β1 and α6β1 activity, enhances expression and phosphorylation of FAK and paxillin, and subsequently leads to cytoskeletal rearrangement and changes of cell morphology [37-39]. Also, it is believed that CD147 plays a role in mediating epithelial-mesenchymal transition (EMT) in the process of HCC progression, providing a slight clue to the function of CD147 in cytoskeleton rearrangement [40]. Recently, we reported that CD147 promotes cell motility by regulating Annexin A2-activated RhoA [5], enhances Src activity and promotes mesenchymal-type cell movement by up-regulating DOCK8 [22]. All these results, together with our data reported here, suggest that CD147 regulates cytoskeleton rearrangement and cell movement at multiple levels and through different mechanisms, although how to coordinate these mechanisms during regulating cell movement at spatio-temporal level still awaits further investigation and it is still difficult to dissect to what extent each mechanism contributes to regulation of cell movement under diverse circumstances. Above all, targeting CD147 could be a promising strategy to reduce migration and metastasis of tumor cells.

## MATERIALS AND METHODS

### Antibodies and biosensors

Antibodies specific for CD147 (sc-9754), α-tubulin (sc-8035), WAVE2 (sc-10392), Annexin A2 (sc-48397), p-Annexin A2 (sc-135753), RhoA (sc-418) and donkey anti-goat IgG-FITC (sc-2024) were purchased from Santa Cruz (Dallas, Texas); p-Src (Tyr416) (2013), p-myosin light chain 2 (Thr18/Ser19) (3674), Src (2109), β-catenin (8814) and myosin light chain 2 (3672) antibodies were purchased from Cell Signaling Technology (Boston, MA); DOCK3 antibody (orb156627) was obtained from Biorbyt (Cambridge, UK); and Alexa 594-conjugated goat anti-mouse IgG and Alexa Fluor 488 phalloidin were purchased from Invitrogen (Carlsbad, CA). Rac1 antibody (ARC03) was obtained from Cytoskeleton (Denver, CO). The Src kinase inhibitor Src I-1 (Sigma, St. Louis, MO) was used at 300 nM. The GSK-3β inhibitor TWS119 (sc-221694, Santa Cruz, Texas) was used at 5 μM. The RhoA biosensor was obtained from Addgene. The Rac1 biosensor was a gift from Dr. Louis Hodgson of Yeshiva University.

### Tissue specimens and IHC analysis

24 tissue specimens of HCC were collected from the Department of Hepatobiliary & Pancreas Surgery, Xijing Hospital, which is affiliated with the Fourth Military Medical University (FMMU) from 2008 to 2009 and were histological confirmed by staining with hematoxylin and eosin (H&E). All individuals provided written informed consent, and the study was approved by the hospital Ethics Committee. For IHC analysis, sections were incubated with primary antibodies and developed with the Histostain^®^-Plus Kit (Invitrogen, Carlsbad, CA). The expression level was independently evaluated by 2 senior pathologists according to the proportion and intensity of positive cells. The following criteria were used to score each specimen: 0 (no staining), 1 (any percentage with weak intensity or < 30% with intermediate intensity), 2 (> 30% with intermediate intensity or < 50% with strong intensity) or 3 (> 50% with strong intensity).

### Cell lines

Human SMMC-7721 hepatoma cell line was obtained from Chinese Academy of Medical Sciences. HuH-7 cells were obtained from the Japanese Collection of Research Bioresources. HepG2, A549 and HCT-8 cells were obtained from the American Type Culture Collection. All cell lines were routinely cultured using standard protocols. A CD147-knockout SMMC-7721 cell line (K-7721) was established using the zinc finger nuclease (ZFN)-targeting approach [41]. Cell line authentication was assessed using short tandem repeat (STR) DNA profiling method in our laboratory and the latest verification was done in March 2014.

### RNA interference

Cells were transfected with a pool of siRNAs using HiPerFect Transfection Reagent according to the manufacturer's instruction (Qiagen). siRNA mixes targeting Annexin A2, CD147, DOCK3, WAVE2 or β-catenin were designed and synthesized by Shanghai GenePharma Co. (Shanghai, China). Silencer negative control siRNA (siCtrl) was used as a negative control under similar conditions. shRNA set (4 individual hairpins) against Annexin A2 and shRNA set (2 individual hairpins) against CD147 were obtained from Shanghai Genechem Co. (Shanghai, China) and used to generate lentiviral particles in packaging cells. The pre-made negative control shRNA served as negative control.

### Generation of CD147 knockout cell lines with the CRISPR/Cas9 system

CD147 knockout HuH-7 and HCT-8 cell lines were prepared by Beijing Biocytogen Co. Ltd. using EGE^TM^ system. Part of CD147 coding region (from 3′ part of exon 2 to exon 8) was replaced with puromycin resistant genes flanked by 5′ and 3′ homologous arm (∼1kb each) respectively, in the donor plasmids. Cas9/sgRNA plasmid was designed against exon 2 of the human CD147 gene. The sgRNA sequence is ggagctgcactgcgaggccgtgg. Primers for amplifying 5′ homologous arm: forward primer 5′-tggcgctgccaaaacttgtcaactgcgc-3′, reverse primer 5′-cctctgctgggacagcggcgcctgga-3′. Primers for amplifying 3′ homologous arm: forward primer 5′-ctgggaggtgggtccagtctgag-3′, reverse primer 5′-gcccaaaacatgccgagaggagtaaca-3′. A total of 1×10^6^ cells were transfected by electroporation (10 μg plasmid/1×10^6^ cells) and then plated on 100mm culture dishes. 3 days after transfection, puromycin (1 μg/ml for HCT-8 and 3 μg/ml for HuH-7) was added to the culture medium, to allow single cell-derived colony formation. After puromycin selection, the survived clones were picked and expanded for genotyping.

### Quantitative real-time PCR analysis

Quantitative real-time PCR analysis was performed as described previously [22]. Primer sequences were listed in Table 2.

**Table 2:** Primer sequences used in Quantitative Real-time PCR

DOCK1	Forward	5′-ACCGAGGTTACACGTTACGAA-3′
DOCK1	Reverse	5′-TCGGAGTGTCGTGGTGACTT-3′
DOCK2	Forward	5′-AGCACAAAATGTTACAGGGCA-3′
DOCK2	Reverse	5′-AGCACAAAATGTTACAGGGCA-3′
DOCK3	Forward	5′-TATGCAGCTTTCGAGGATCTGT-3′
DOCK3	Reverse	5′-GCCCATTCTTGTAGAGTTGCT-3′
DOCK4	Forward	5′-GGATACCTACGGAGCACGAG-3′
DOCK4	Reverse	5′-AGCCATCACACTTCTCCAGG-3′
DOCK5	Forward	5′-CTGTAGCAGCCTTAGTCGCC-3′
DOCK5	Reverse	5′-GCAAGGAGAGCTCCACATCT-3′
DOCK6	Forward	5′-TGTATGATGTGCGGGAGAAAAA-3′
DOCK6	Reverse	5′-AGGGGTAGGTCACAGAGAAGA-3′
DOCK7	Forward	5′-TGGGCCTGTAGTATCTTTGACT-3′
DOCK7	Reverse	5′-TCTTCCTCATCTGGTGATGGAT-3′
DOCK8	Forward	5′-ACGCGCCGTGTAACTGTGAA-3′
DOCK8	Reverse	5′-CCCCGAGCTCCTGGGCAA-3′
DOCK9	Forward	5′-GCTTCCGAACAAAGTGGTCAA-3′
DOCK9	Reverse	5′-GCCATGCTTGGTGATCCCA-3′
DOCK10	Forward	5′-CCGTATGCCTTTTGCTTGGG-3′
DOCK10	Reverse	5′-GCTTATTCTGTCGGCCCTTCT-3′
DOCK11	Forward	5′-AGAGGCTGCGATGTGTTATGT-3′
DOCK11	Reverse	5′-CTCATAAATTGGAACGATCAACT-3′
WAVE2	Forward	5′-CTTTCAGCCATCCGTCAAGG-3′
WAVE2	Reverse	5′-AATCGGACCAGTCGTCCTCA-3′
Annexin A2	Forward	5′-GAGGATGGCTCTGTCATTGATT-3′
Annexin A2	Reverse	5′-CTGGTAGTCGCCCTTAGTGTCT-3′
CD147	Forward	5′-ACTCCTCACCTGCTCCTTGA-3′
CD147	Reverse	5′-GCCTCCATGTTCAGGTTCTC-3′
GAPDH	Forward	5′-GCACCGTCAAGGCTGAGAAC-3′
GAPDH	Reverse	5′-TGGTGAAGACGCCAGTGGA-3′

### Nuclear/cytoplasmic fractionation

Nuclear/cytoplasmic fractionation was performed using NE-PER™ Nuclear and Cytoplasmic Extraction Reagents (Pierce, US) according to the manufacturer's instructions.

### *In vitro* wound-healing assay

*In vitro* wound-healing assay was performed as described previously [29]. Briefly, 24 h after treatment, the cells were harvested and seeded in 12-well plates until confluent. A pipette was used to scratch the monolayer. The cells were then washed with serum-free medium. Photomicrographs were obtained at various time points (0h and 24h), and the relative migration distance was calculated using the following formula: the relative migration distance (%) = 100 (AX-BX)/(A blank - B blank), where A is the width of the cell wound before incubation, and B is the width of the cell wound after incubation.

### FRET assay

The FRET assay was performed according to the methodology described previously [42-45]. Briefly, three sequential images were acquired with suitable filter sets for donor (EGFP; excitation at 488 nm and emission at 515 nm), acceptor (DsRed; excitation at 543 nm and emission at 585 nm), and FRET (excitation at 488 nm and emission at 585 nm) under an oil immersion objective (n.a. = 1.40). Donor and acceptor images were utilized to generate CoA and CoB data for the next FRET calculation with NIS-Elements software (Nikon, Japan). The FRET efficiency calculation and image procession were performed with analysis software (Nikon, Japan) [46].

### Fluorometric analysis of the RhoA/Rac1 activation

Cells were prepared as previously reported [47]. Images were obtained using an A1 confocal microscopy (Nikon, Japan). For emission ratio imaging, the filter sets were used as previously reported [48]. NIS-Elements software (Nikon, Japan) was used to analyze the images following previously described methods [47]. Briefly, images were dark-current and background-subtracted. Binary masks generated through intensity thresholding were applied to each emission channels, and the matched FRET and donor image sets were ratioed to depict Rac1 or RhoA activation throughout the cell. A linear pseudocolour lookup table was applied.

### *In vitro* kinase assay

The his_6_-tagged proteins were purified as described previously [49]. Purified baculovirus-expressed human Src was obtained from Millipore. The kinase assay was performed as described previously [50]. Briefly, purified Annexin A2 and purified Src were incubated with or without purified CD147, C2 domain or I domain in kinase buffer (50mM HEPES pH 7.8, 5mM MgCl_2_, 150mM NaCl, 1mM DTT, 1mM ATP) plus 1mM sodium orthovanadate at 30°C for 30 minutes. The reactions were stopped with the addition of Laemmli sample buffer before subjected to gel electrophoresis and Western blot.

### Surface plasmon resonance (SPR)

SPR studies were performed using a BioRad ProteOn XPR36 instrument according to a One-shot Kinetics protocol [51, 52]. GLC chips were initialized using glycerol and preconditioning was performed by sequential injections of 0.5% SDS, 100 Mm HCl, and then 50 mM NaOH, each for 60 sec at 25 μl/min. The surface was then activated using 40 mM EDAC and 10 mM Sulfo-NHS, injected for 5 min at 25 μl/min. 50 μg/ml of Annexin A2 in 10 mM sodium acetate, pH 5.5, was injected in the vertical direction over the desired channels for 6 min at 25 μl/min. 50 μg/ml of BSA was also immobilized in the same way using as a control. The remaining activated carboxyl groups were blocked with a 3 min injection of 1 M ethanolamine at 25 μl/min in the vertical direction. The chip was washed with PBS until a stable baseline was achieved. The running buffer was then switched to TBS with 0.05‰ Tween-20 (TBS-T). A concentration series of the analyte of interest was injected over the immobilized ligand (Annexin A2). Injection of the analyte diluted in TBS-T was done in the horizontal direction at 50 μl/min for 3 min. An equivalent buffer injection was used for reference subtraction. The dissociation time was set to 12 min. Equilibrium and rate constants were calculated using ProteOn Manager Software. A local R_max_ value and the Langmuir model were used.

### Immunofluorescence

Immunofluorescence was performed as described previously [22]. Briefly, cells were harvested and allowed to attach for 24 h to fibronectin-pre-coated cell culture dishes with glass bottoms (801002, NEST Biotechnology Co., LTD.). After washing twice with PBS, the cells were fixed in paraformaldehyde in PBS, permeabilized with 0.1% Triton X-100, and blocked with 1% BSA in PBS for 1 h. The dishes were first incubated with the indicated antibodies for 1 h, washed twice with PBS, and then incubated with Alexa 488-phalloidin solution and the corresponding FITC-conjugated secondary antibodies for 30 min in the dark. Cell nuclei were dyed with DAPI (Vector Labs). After washing, the cells were visualized using an A1R-A1 confocal laser microscope system (Nikon, Japan).

### Live-cell imaging

For live-cell imaging [53], cells (1×10^4^) were harvested at 24 h after transfection and allowed to attach for 12 h to fibronectin-pre-coated 35 mm dishes. Time-lapse images using phase-contrast and fluorescence microscopy (A1R-A1 confocal laser microscope system, Nikon) were taken at 2 min intervals and assembled into movies using NIS-Elements software (Nikon, Japan). For quantitative image analysis of the lamellipodial extension after transfection of siDOCK3 or siCtrl, we took measurements of cell area changes by subtracting the areas of the cells at 0 min from those at 2 min using NIS-Elements software (Nikon).

### *In vivo* metastasis assay

Male BALB/c nude mice at 4 to 6 weeks of age were provided by the Laboratory Animal Research Center of FMMU, and the animal study was approved by the Animal Care and Use Committee of FMMU. The experimental metastatic potential of HCC cells was assessed following intrasplenic injection as described previously [40].

## SUPPLEMENTARY MATERIAL FIGURE AND MOVIES






