# Supplementary material for: CD147 regulates cancer migration via direct interaction with Annexin A2 and DOCK3-β-catenin-WAVE2 signaling
Source: Oncotarget. 2015 Dec 22;7(5):5613–29. doi: 10.18632/oncotarget.6723 (PMC4868709; doi:10.18632/oncotarget.6723)
Supplement: Supplementary file 1 [file oncotarget-07-5613-s001.pdf]

CD147 regulates cancer migration *via* direct interaction with Annexin A2 and DOCK3-β-catenin-WAVE2 signaling

Supplementary Material

ESM_1 Quantification of p-Annexin A2 normalized to levels of CD147 and p-Src expression. Data represent average ± SD; ***$p<0.001$, **$p<0.01$, ns $p>0.05$ by ANOVA. These analyses correspond to the image shown in Fig. 2e

ESM_2-3 DOCK3 suppresses lamellipodium dynamics. Movie ESM_2 shows that lamellipodium dynamics was enhanced in cells transfected with siDOCK3. Cells transfected with siCtrl were used as control (ESM_3). These movies correspond to the images shown in Fig. 4i

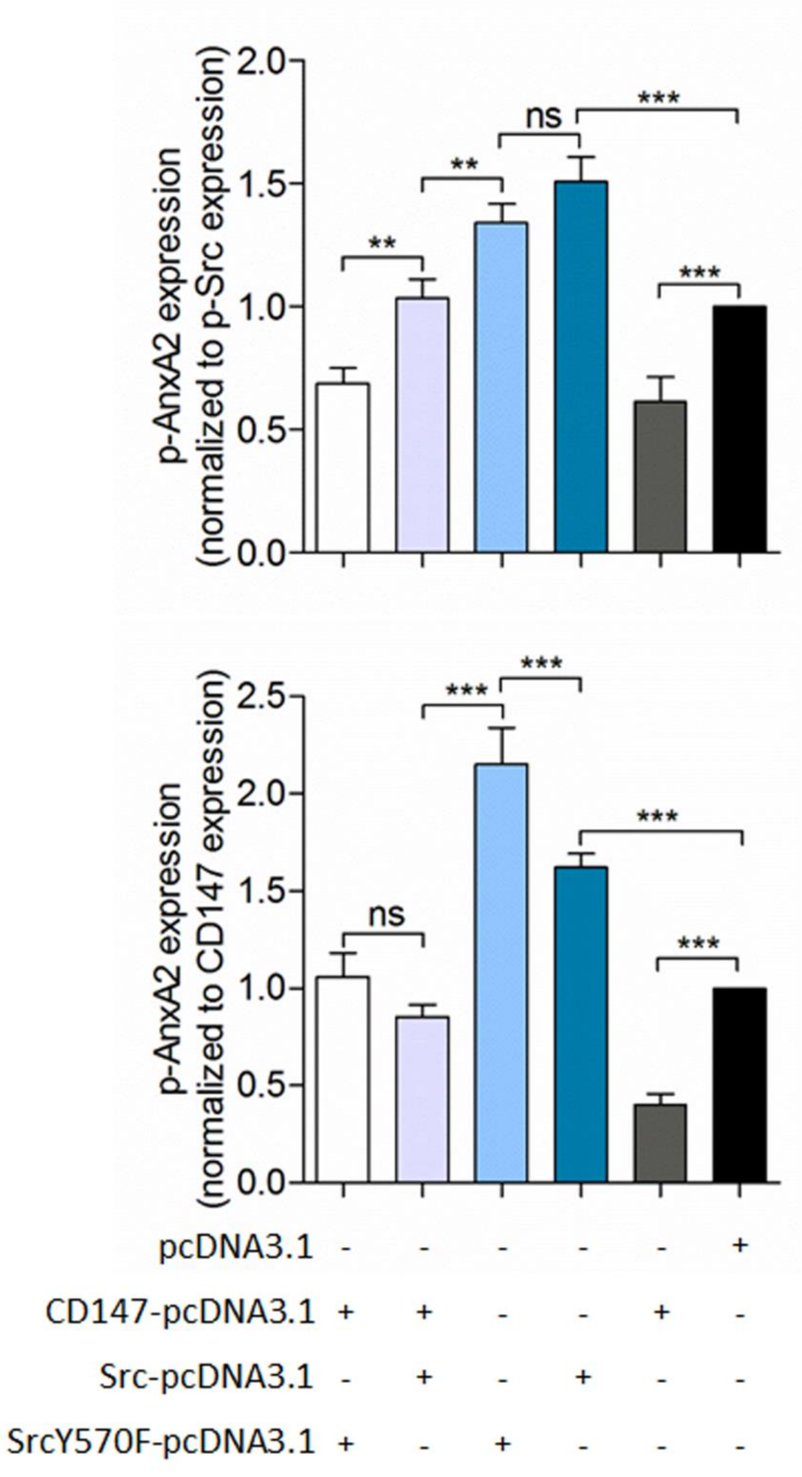

p-AnxA2 expression
(normalized to p-Src expression)
2.0
1.5
1.0
0.5
0.0
**
**
ns
***
***
p-AnxA2 expression
(normalized to CD147 expression)
2.5
2.0
1.5
1.0
0.5
0.0
ns
***
***
***
***
pcDNA3.1 - - - - - +
CD147-pcDNA3.1 + + - - + -
Src-pcDNA3.1 - + - + - -
SrcY570F-pcDNA3.1 + - + - - -
